# Maximum Standard Uptake Value of Pre‐Therapeutic ^18^F‐Fluorodeoxyglucose Positron Emission Tomography Predicts Outcomes in Indolent Adult T‐Cell Leukemia/Lymphoma Patients With Cutaneous Involvement

**DOI:** 10.1111/1346-8138.17867

**Published:** 2025-08-06

**Authors:** Kyoko Nogami, Yotaro Nishikawa, Kosuke Mochida, Tamasa Terada, Minako Azuma, Michikazu Nakai, Masahiro Amano

**Affiliations:** ^1^ Department of Dermatology University of Faculty Miyazaki of Medicine Miyazaki Japan; ^2^ Department of Radiology University of Faculty Miyazaki of Medicine Miyazaki Japan; ^3^ Clinical Research Support Center University of Miyazaki Hospital Miyazaki Japan

**Keywords:** ATLL, cutaneous involvement, FDG‐PET, retrospective study, SUVmax

## Abstract

The prognostic utility of 18F‐fluorodeoxyglucose positron emission tomography (FDG‐PET) in adult T‐cell leukemia/lymphoma (ATLL) remains unclear, particularly in patients with indolent subtypes and cutaneous involvement. This study aimed to evaluate the usefulness of FDG‐PET in predicting clinical outcomes in patients with indolent ATLL presenting with skin lesions. We retrospectively reviewed indolent ATLL with cutaneous involvement who underwent ^18^F‐fluorodeoxyglucose positron emission tomography at our institute for initial disease staging between April 2007 and March 2022. The data obtained were compared with the findings of cutaneous involvement in ATLL. The effect of maximum standardized uptake value on progression‐free survival was analyzed using the Kaplan–Meier method. Patients were divided into groups based on whether their maximum standardized uptake value was above or below the overall mean maximum standardized uptake value (2.18), and progression‐free survival was compared between the groups. Forty‐three patients with indolent ATLL were included. We divided the cutaneous involvement of ATLL into six subtypes according to a previously reported classification of eruption types: patch, plaque, multipapular, nodulotumoral, erythrodermic, and purpuric. A total of 18 of 43 patients (41.9%) had ^18^F‐fluorodeoxyglucose‐positive cutaneous lesions. However, 25 patients showed no ^18^F‐fluorodeoxyglucose uptake in cutaneous lesions. There was a significant difference in the mean maximum standardized uptake value between the nodulotumoral and multipapular (*p* = 0.036), nodulotumoral and patch (*p* = 0.036). There was a statistically significant difference in progression‐free survival between the maximum standardized uptake value ≥ 2.18 and < 2.18 groups (*p* = 0.020). These findings indicate that the maximum standardized uptake value in cutaneous lesions could determine the prognostic association of ATLL with cutaneous lesions. Careful follow‐up is required for patients with a higher maximum standardized uptake value for cutaneous lesions.

## 1 Introduction

Adult T‐cell leukemia/lymphoma (ATLL) is caused by human T‐cell lymphotropic virus type 1 (HTLV‐1) [1] and is endemic in Southwest Japan and the Caribbean basin [2]. There are several subtypes of ATLL: acute, lymphoma, chronic, and smoldering [3]. Patients with the chronic type have been further divided into two categories based on the presence or absence of either of three unfavorable prognostic factors: serum blood urea nitrogen or lactate dehydrogenase levels above the normal upper limit, or albumin levels below the normal lower limit. Chronic ATLL without unfavorable prognostic factors is known as favorable chronic ATLL, and along with the smoldering type, it is referred to as indolent ATLL. Chronic ATLL with unfavorable prognostic factors is known as unfavorable chronic ATLL.

Indolent ATLL has a better prognosis than the aggressive type. The median survival times for favorable chronic types and smoldering types were not reached (estimated to be ≥ 40.7 months) and 55.0 months, respectively [4]. Acute, lymphoma, and unfavorable chronic types of ATLL are classified as aggressive ATLL, with median survival times of 8.3, 10.6, and 27 months, respectively [4].

Some indolent ATLL cases can progress to aggressive ATLL, that is, acute transformation.

ATLL commonly involves the skin, affecting 43%–72% of patients [5]. Patients with ATLL often present with nodules/tumors (34.7%), erythematous plaques (22.6%), and erythematous papules (19.4%), similar to other cutaneous T‐cell lymphomas (CTCL) [6]. In 2011, Sawada et al. [7] classified ATLL into six major subtypes: patch, plaque, multipapular (MP), nodulotumoral (NT), erythrodermic, and purpuric. The NT subtype has an inferior prognosis compared to the other subtypes, the MP subtype has an intermediate prognosis, and the plaque and patch subtypes have better prognoses than the other subtypes.

A prognostic index for indolent ATLL (iATL‐PI) has been previously reported as a prognostic parameter for chronic and smoldering ATLL, with soluble interleukin‐2 receptor (sIL‐2R) levels as an independent prognostic factor [8]. Although several genomic variants have been reported as predisposing factors for the acute transformation of indolent ATLL [9], predicting this transformation in general practice remains difficult. One possible method for predicting the prognosis of indolent type ATLL is ^18^F‐fluorodeoxyglucose (FDG)‐positron emission tomography (PET). FDG‐PET improves the accuracy of lymphoma staging for Hodgkin lymphoma, non‐Hodgkin lymphoma [10], and natural killer (NK)/T‐cell lymphomas [11]. However, there are only a few reports describing the FDG‐PET imaging of ATLL and even fewer with the imaging of cutaneous involvement in ATLL.

FDG uptake above the blood pool is considered abnormal, and the highest standardized uptake value is defined as the maximum standardized uptake value (SUVmax). In mature T‐cell and NK cell malignancies, the SUVmax varies widely among patients with the same histologic subtype [12]. This variation in FDG uptake could be explained by the histological profile, for example, the proliferating nature of tumor cells or the proportion of viable tumor cells and reactive non‐malignant cells [13, 14].

FDG‐PET provides physiological and anatomical information on a wide spectrum of nodal and extranodal lesions in malignant lymphomas, and it plays an important role in the staging and follow‐up of Hodgkin lymphoma and non‐Hodgkin lymphoma [10]. However, only a few reports on cutaneous imaging‐based findings in CTCL have been published. Few studies have also been reported on the usefulness of FDG‐PET in ATLL.

Here, we retrospectively reviewed the results of FDG‐PET in patients with ATLL diagnosed according to the Shimoyama classification [3]. We analyzed the positive rate and SUVmax of pretreatment FDG‐PET in patients with cutaneous involvement of ATLL to define the role of FDG‐PET in predicting clinicopathological features and outcomes.

## 2 Methods

### 2.1 Study Design

In this retrospective study, patients with ATLL who underwent FDG‐PET as part of their initial staging were recruited at our institute between April 2007 and March 2022. All patients were diagnosed with ATLL on the basis of the Shimoyama classification, which includes acute, lymphoma, chronic, and smoldering types [3]. We excluded aggressive ATLL types and selected only indolent types, including smoldering and favorable chronic ATLL.

Disease progression was defined as the acute transformation of indolent ATLL to aggressive ATLL. FDG‐PET was used to assess the cutaneous lesions and the absence of metastatic lesions in the internal organs and lymph node involvement. The criteria for cutaneous ATLL lesions were defined as follows: (1) histologically and/or cytologically proven lymphoid malignancy with positive T‐cell surface antigens (CD3, 4, and 25), (2) abnormal T‐lymphoid cells in the skin tissue, and (3) antibody to HTLV‐I present in sera at diagnosis.

We categorized ATLL skin lesions into six subtypes based on the criteria for ATLL‐related skin involvement: patch, plaque, MP, NT, erythrodermic, and purpuric according to the criteria for categorizing ATLL‐related skin involvement in a previous study by Sawada et al. [7].

Progression‐free survival (PFS) was defined as the time from the date of the first FDG‐PET to the date of disease progression or death due to any cause, whichever occurred first. Patients who had not experienced progression or death by the end of the observation period (April 17, 2023), as well as those who were lost to follow‐up before progression or death during the observation period, were treated as censored.

### 2.2 Histopathological Analysis

We examined the histopathological specimens to determine the extent of abnormal lymphocyte infiltration across the different skin layers. In addition, the clonal integration of HTLV‐1 proviral DNA was confirmed in the cutaneous lesions of a subset of cases. All patients were diagnosed with ATLL by multiple pathologists and dermatologists.

### 2.3 FDG‐PET Study

All patients with previously untreated and histologically proven ATLL who were evaluated using FDG‐PET at initial diagnosis were eligible for the study. We performed FDG‐PET/computed tomography (CT) using a whole‐body PET/CT system. All patients were identified as having no hyperglycemia or no diabetes and fasted for 4 h before examination.

A nuclear medicine physician blinded to the patients' clinical outcomes independently reviewed all FDG‐PET scans. In qualitative evaluation, FDG uptake above the blood pool was considered abnormal. The SUVmax was recorded for quantitative analysis and recorded in patients with FDG‐avid lesions with the value of ≥ 1. To evaluate diagnostic accuracy, we compared the PET findings for lesion detection with skin biopsy results. PET‐positive sites were considered true positives when confirmed by skin biopsy results and false positives, whereas PET‐negative sites were considered true negatives when verified by biopsy results and false negatives.

### 2.4 Statistical Analyses

Data are expressed as median and interquartile range (IQR) for continuous variables and as percentage (%) for categorical variables. Continuous variables were compared using the Wilcoxon rank‐sum test, whereas categorical variables were compared using the chi‐square test. Comparisons of SUVmax values by ATLL type were performed using the Steel method.

Univariate and multivariate survival analyses for PFS between SUVmax‐defined groups (≥ 2.18 vs. < 2.18) were conducted using the Kaplan–Meier method with the log‐rank test and the Cox proportional hazards model to estimate hazard ratios (HRs) and 95% confidence intervals (CIs).

Multivariate analysis was conducted with PFS as the dependent variable and SUVmax and iATL‐PI as independent variables. The indolent ATL prognostic index (iATL‐PI) was calculated based on serum soluble interleukin‐2 receptor (sIL‐2R) levels. Patients were categorized into three risk groups according to sIL‐2R cut‐off values: low‐risk (sIL‐2R ≤ 1000 U/mL), intermediate‐risk (sIL‐2R 1000–6000 U/mL), and high‐risk (sIL‐2R > 6000 U/mL) [9]. Statistical analyses were performed using STATA18 (StataCorp; College Station, TX, USA), with a significance threshold of *p* < 0.05.

### 2.5 Ethics Statement

The ethics committee of the University of Miyazaki, Japan approved this study.

## 3 Results

### 3.1 Patient Characteristics

Patient characteristics are presented in Table 1. The mean SUVmax for all measured values was 2.18, and the values were categorized into groups above and below the mean. There were 33 patients with a SUVmax < 2.18 and 10 patients with a SUVmax ≥ 2.18. Statistical analysis showed no significant differences in age, sex, or blood test results between the two groups (*p* > 0.05).

**TABLE 1:** Patient characteristics at diagnosis.

Factor	Level	Value	SUVmax < 2.18	SUVmax ≥ 2.18	*p* value
*N*		43	33	10	
Sex	Male	27 (63%)	20 (61%)	7 (70%)	0.59
Age, median (IQR)		69 (59, 76)	71 (60, 75)	64 (59, 81)	0.99
WBC, median (IQR)		6700 (5300, 8100)	7000 (6000, 8100)	5650 (5100, 8100)	0.36
Total lymphocyte count, median (IQR)		1850 (1305.6, 2380)	1950 (1537.5, 2640)	1374.3 (1173, 1696)	0.070
Abnormal lymphocyte (%), median (IQR)		1 (0, 3)	1 (0, 4)	1.5 (0.5, 2)	0.91
LDH, median (IQR)		205 (175, 246)	205 (175, 246)	217 (175, 292)	0.82
Corrected calcium (mg/dL), median (IQR)		9.4 (9.1, 9.7)	9.4 (9.1, 9.7)	9.35 (8.8, 9.7)	0.55
sIL‐2R, median (IQR)		1010 (742, 1520)	1010 (742, 1600)	986.5 (513, 1380)	0.64
skin ATLL type	MP	7 (16%)	7 (21%)	0 (0%)	0.022
	NT	10 (23%)	4 (12%)	6 (60%)	
	Erythroderma	2 (5%)	2 (6%)	0 (0%)	
	Patch	6 (14%)	6 (18%)	0 (0%)	
	Plaque	15 (35%)	11 (33%)	4 (40%)	
	Purpuric	3 (7%)	3 (9%)	0 (0%)	
iATL‐PI	Low	20 (46.51%)	15 (45.45%)	5 (50.0%)	
	Middle	20 (46.51%)	15 (45.45%)	5 (50.0%)	
	High	1 (2.33%)	1 (3.03%)	0	
	Missing	2 (4.65%)	2 (6.06%)	0	

Abbreviations: iATL‐PI: indolent ATL prognostic index; ATLL : adult T‐cell leukemia/lymphoma; IQR : interquartile range; LDH: lactate dehydrogenase; MP: Multipapular subtype; NT: nodulotumoral subtype; sIL‐2R: soluble interleukin‐2 receptor; SUVmax : standardized uptake value; WBC: white blood cell count.

**FIGURE 1:**
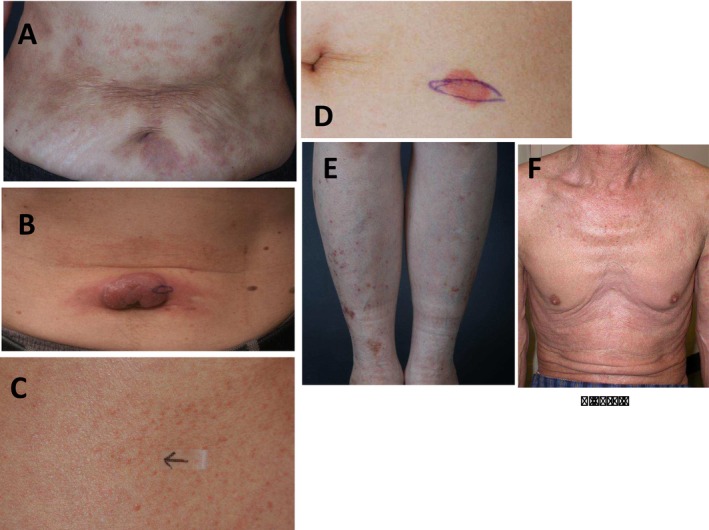
Clinical appearance. (A) Patient number (no.) 4 was diagnosed as having smoldering type adult T‐cell leukemia/lymphoma (ATLL) and patch subtype. (B) Patient no. 42 was diagnosed as having smoldering type ATLL and nodulotumoral subtype. (C) Patient no. 2 was diagnosed as having smoldering type ATLL and maculopapular subtype. (D) Patient no. 16 was diagnosed as having smoldering type ATLL and plaque subtype. (E) Patient no. 17 was diagnosed as having smoldering type ATLL and purpuric subtype. (F) Patient no. 24 was diagnosed as having smoldering type ATLL and erythrodermic subtype.

The diagnoses included favorable chronic ATLL (*n* = 1) and smoldering ATLL (*n* = 42). Cutaneous lesions were multiple and generalized in most patients. The patch, plaque, MP, NT, purpuric, and erythrodermic subtypes were found in 6, 15, 7, 10, 3, and 2 patients, respectively (Figure 1).

### 3.2 Pathological Findings

Clonal integration of HTLV‐1 proviral DNA was confirmed in 24 cutaneous lesions, whereas it was not detected in six cases.

The depth of infiltration of abnormal lymphocytes confirmed by skin biopsy showed that 29 cases had infiltration up to the superficial dermis, 9 had infiltration extending from the entire dermis to part of the subcutaneous adipose tissue, and 4 had tumor cells replacing the full thickness of the skin. In one case, the biopsy specimen was unavailable and could not be evaluated. All three full‐thickness cases were of the NT subtype, whereas all the patch subtypes were superficial.

### 3.3 FDG‐PET Analysis

**FIGURE 2:**
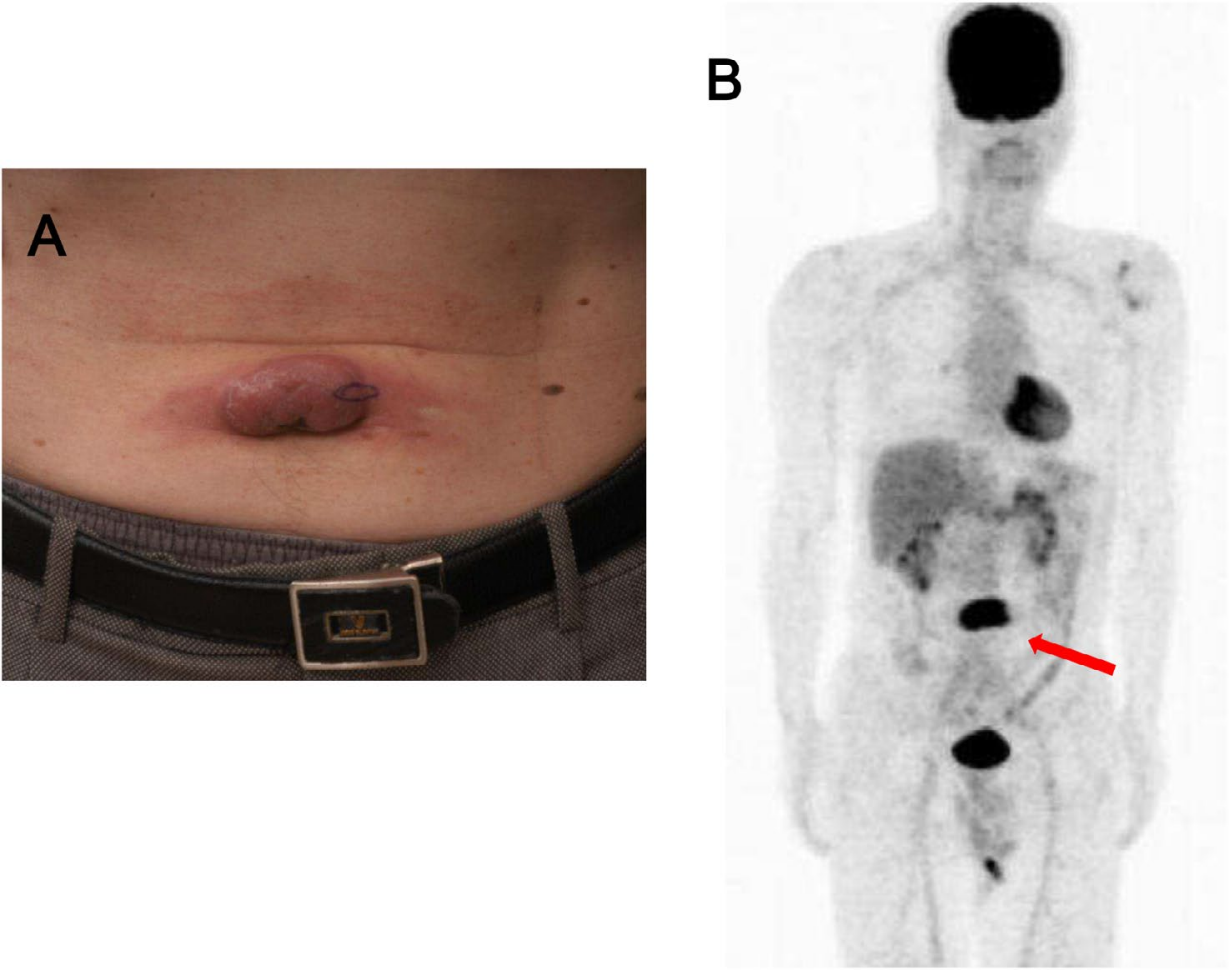
(A) Clinical appearance of patient No. 42. (B) 18F‐fluorodeoxyglucose (FDG) positron emission tomography (PET) imaging shows FDG uptake in the lesion (arrow).

Initial FDG‐PET was performed in all 43 patients and showed abnormal cutaneous FDG uptake in only 18 (41.9%) (Figure 2). The SUVmax was recorded in patients with FDG‐avid lesions with a value of ≥ 1 and varied widely among patients with ATLL (Table S1; patient numbers 26–43, total 18 patients). At the time of diagnosis, FDG‐PET could not detect cutaneous lesions in 25 patients.

**FIGURE 3:**
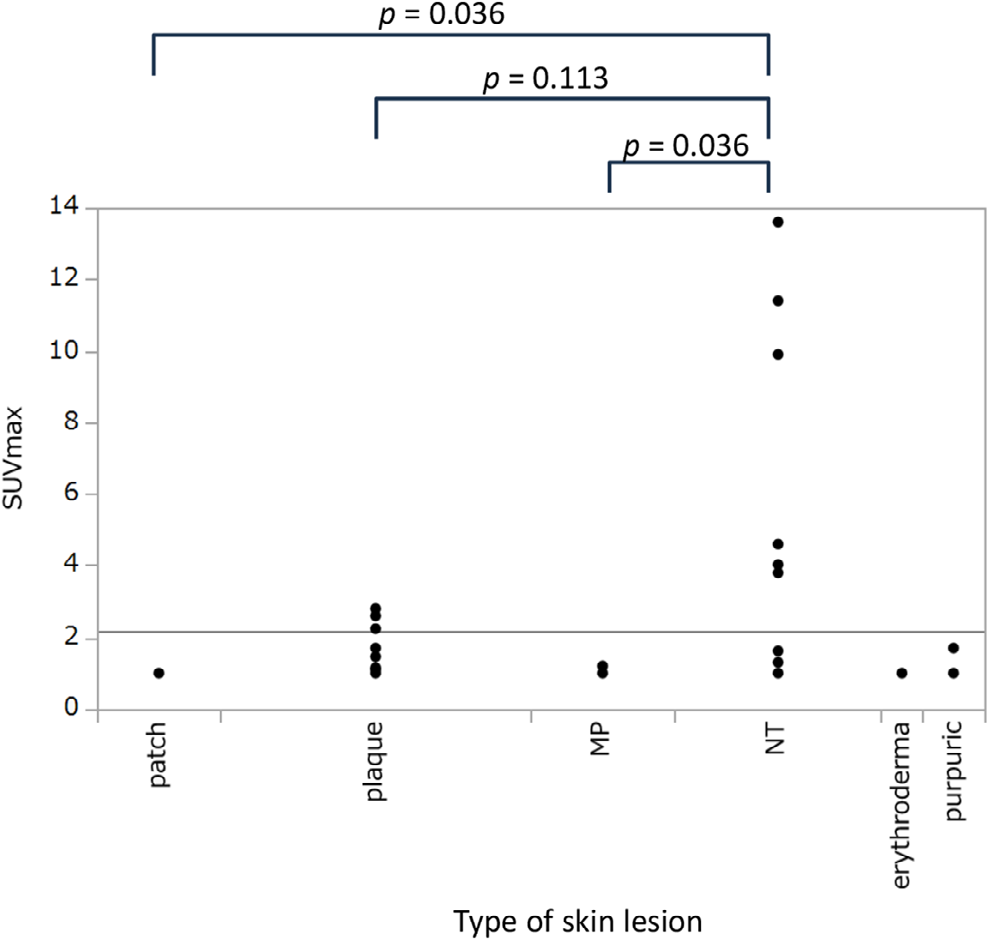
Maximum standardized uptake value (SUVmax) of cutaneous lesions according to adult T‐cell leukemia/lymphoma (ATLL) subtypes. The mean SUVmaxs are 1.03, 1.51, 5.225, 1.0, and 1.233 for the multipapular (*n* = 7), plaque (*n* = 15), nodulotumoral (*n* = 10), patch (*n* = 6), and purpura subtypes, respectively. Comparisons of the SUVmax by ATLL type were performed using the Steel method.

Among the patients, the mean SUVmaxs were 2.18, 1.03, 1.51, 5.225, 1.0, 1.233, and 1.0 for all subtypes (*n* = 43) and the MP (*n* = 7), plaque (*n* = 15), NT (*n* = 10), patch (*n* = 6), purpura (*n* = 3), and erythrodermic subtypes (*n* = 2), respectively (Figure 3). The mean SUVmax was compared between the groups for each subtype. There was a statistically significant difference in the mean SUVmax between the NT and MP (*p* = 0.036)and the NT and patch (*p* = 0.036).

### 3.4 Clinical Outcomes

During the observation period, 17 of the 43 patients developed aggressive ATLL, with a median follow‐up duration of 1054.5 days (IQR, 37–5731 days).

We divided the patients into two groups based on the mean SUVmax value (SUVmax ≥ 2.18 vs. < 2.18).

**FIGURE 4:**
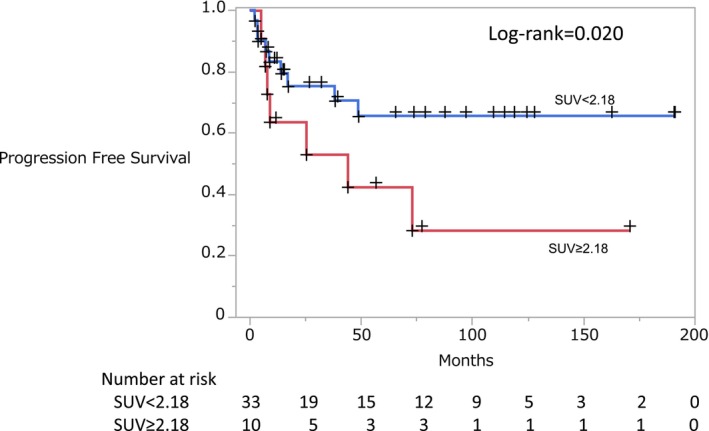
Overall survival rates of patients with standardized uptake values (SUVmaxs) for adult T‐cell leukemia/lymphoma. For Kaplan–Meier survival curve comparisons, *p* values were calculated using the log‐rank test. A statistically significant difference in PFS is observed between the SUVmax ≥ 2.18 and SUVmax < 2.18 groups (*p* = 0.020, log‐rank test). Censored observations are indicated by plus signs (+).

The Kaplan–Meier method was used to analyze the effect of SUVmax on progression‐free survival (PFS). The corresponding survival curves are presented in Figure 4. A statistically significant difference in PFS was observed between the SUVmax ≥ 2.18 and < 2.18 groups (log‐rank test, *p* = 0.020, Figure 4).

Univariate Cox regression survival analysis showed that only SUVmax was significantly associated with PFS.

Based on these findings and previous reports identifying iATL‐PI as a prognostic marker, both SUVmax and iATL‐PI were included in the multivariate Cox regression analysis. SUVmax remained an independent prognostic factor for PFS after adjustment for iATL‐PI (Table 2).

**TABLE 2:** Univariate and multivariate analyses of prognostic factors for progression‐free survival (PFS) in indolent ATLL with cutaneous lesions, based on Cox proportional hazards models.

Variable	HR (95% CI) univariate	*p* value univariate	HR (95% CI) multivariate	*p* value multivariate
SUVmax				
SUVmax < 2.18 (ref)	1.00	—	1.00	—
SUVmax ≥ 2.18	3.06 (1.14, 8.24)	0.026	2.74 (1.01, 7.41)	0.047
Sex				
Female (ref)	—	—	1.00	—
Male	0.94 (0.34, 2.58)	0.897	—	—
**Age**	1.04 (0.99, 1.09)	0.099	—	—
**WBC**	1.00 (0.99, 1.00)	0.498	—	—
**Total lymphocyte count**	0.99 (0.99, 1.00)	0.901	—	—
**Abnormal lymphocyte**	0.99 (0.88, 1.12)	0.891	—	—
**LDH**	1.00 (0.99, 1.01)	0.804	—	—
**Corrected calcium**	0.74 (0.20, 2.74)	0.652	—	—
**sIL‐2R**	1.00 (0.99, 1.00)	0.066	—	—
NT or not NT				
Not NT (ref)	—	—	1.00	—
NT	2.41 (0.83, 6.99)	0.105	—	—
iATL‐PI				
Low (ref)	1.00	—	1.00	—
Middle	1.75 (0.65, 4.71)	0.268	1.60 (0.59, 4.33)	0.354
High	Not determined*	—	Not determined*	—

Abbreviations: iATL‐PI: indolent ATL prognostic index; ATLL : adult T‐cell leukemia/lymphoma; CI : confidence interval; HR : hazard ratio; LDH: lactate dehydrogenase; NT: nodulotumoral subtype; PFS: progression‐free survival; ref: reference group; sIL‐2R: soluble interleukin‐2 receptor; SUVmax: standardized uptake value max; WBC: white blood cell count.

* HR for the high‐risk group is not determined because there was only one patient who did not develop the event in this group.

[Correction added on 18 August 2025 after first online publication: “1.00” was replaced by “—” in the column “HR (95% CI) univariate”.]

## 4 Discussion

All 43 patients in the present study were histologically diagnosed with malignant lymphoma. FDG‐PET revealed cutaneous lesions in 18 patients at the time of diagnosis, including 9 (90%) of 10 NT subtypes and 8 (53.3%) of 15 plaque‐subtype lesions. Additionally, cutaneous lesions were detected in only 1 of 7 MP (14.3%) and 3 purpura subtypes (33.3%) using FDG‐PET. In contrast, FDG‐PET did not detect any lesions in the patch (*n* = 6) or erythroderma (*n* = 2) subtypes. These results suggest that the sensitivity of FDG‐PET varies depending on cutaneous lesion subtype.

Here, we demonstrated that FDG‐PET occasionally reveals skin tumors in patients with the NT subtype. An increase in the SUVmax in patients with the NT subtype may indicate a higher tumor burden. The mean SUVmax was the highest for the NT subtype, and the differences between the NT subtype and the MP, patch, and plaque subtypes were significant. However, owing to the small sample size, erythroderma and purpura subtypes could not be accurately assessed. Additionally, FDG‐PET had poor sensitivity at cutaneous sites in the MP and patch subtypes, probably because the lesions were very small. Valencak et al. [15] reported that low glucose metabolism in early‐stage cutaneous lesions of CTCL may be responsible for these false‐negative results.

FDG‐PET findings of cutaneous lesions differ among reports because FDG avidity is influenced by tumor load and lymphoma subtypes [16, 17]. Schoder et al. [18] reported that the SUVmax is useful in distinguishing between aggressive and indolent lymphomas. The variability of the SUVmax in malignant tissues is related to various factors, including tumor type, proliferation rate, blood supply, and heterogeneity [19]. Several reports have demonstrated the potential prognostic value of SUVmax on pretreatment FDG‐PET in B‐cell lymphoma [20, 21]. Our results of the effect of SUVmax on PFS in 43 patients indicated that patients with an initial FDG‐PET examination showing a SUVmax ≥ 2.18 have worse progression than those with a lower SUVmax. The SUVmax in patients with ATLL and NT lesions was significantly higher than that in patients with the plaque, MP, or erythroderma subtypes. These results are generally consistent with recent findings that the NT subtype of ATLL is associated with unfavorable outcomes [9]. For reasons already stated, cutaneous subtypes in ATLL are important factors in PFS; however, in the univariate analysis, the NT subtype or other subtypes were not statistically significant factors for defining PFS in our study. In addition, because few NT subtypes do not have FDG‐avid lesions, dermatologists and radiologists must pay attention to patients with ATLL in whom FDG is not avid.

In 2017, a prognostic index for smoldering and chronic ATLL was developed by Katsuya et al. [9] Since a retrospective study identified sIL‐2R as an independent prognostic factor, we performed multivariate analyses of sIL‐2R and SUVmax for PFS. Our results suggest that the SUVmax could be a useful prognostic factor regardless of the ATLL skin subtype.

In conclusion, patients with cutaneous lesions of ATLL who showed a SUVmax ≥ 2.18 on initial FDG‐PET scan had a poor prognosis. Thus, patients with ATLL and cutaneous lesions with a high SUVmax, especially the NT subtype, require careful follow‐up.

## Ethics Statement

Approval of the research protocol by an Institutional Review Board: This study was approved by the ethics committee of the University of Miyazaki, Japan.

## Conflicts of Interest

The authors declare no conflicts of interest.

## Supporting information


Table S1.

## Data Availability

Research data are not shared.
